# Comprehensive Behavioral Analysis of Opsin 3 (Encephalopsin)-Deficient Mice Identifies Role in Modulation of Acoustic Startle Reflex

**DOI:** 10.1523/ENEURO.0202-22.2022

**Published:** 2022-09-27

**Authors:** Brian A. Upton, Gowri Nayak, Ivy Schweinzger, Shane P. D’Souza, Charles V. Vorhees, Michael T. Williams, Brian R. Earl, Richard A. Lang

**Affiliations:** 1 The Visual Systems Group, Abrahamson Pediatric Eye Institute, Cincinnati Children’s Hospital Medical Center, Cincinnati, OH 45229; 2 Center for Chronobiology, Division of Pediatric Ophthalmology, Cincinnati Children’s Hospital Medical Center, Cincinnati, OH 45229; 3 Molecular and Developmental Biology Graduate Program, University of Cincinnati, College of Medicine, Cincinnati, OH 45229; 4 Medical Scientist Training Program, University of Cincinnati, College of Medicine, Cincinnati, OH 45267; 5 Department of Communication Sciences and Disorders, University of Cincinnati, College of Allied Health Sciences, Cincinnati, OH 45267; 6 Department of Otolaryngology-Head and Neck Surgery, University of Cincinnati, College of Medicine, Cincinnati, OH 45267; 7 Division of Pediatric Neurology, Cincinnati Children’s Hospital Medical Center, Cincinnati, OH 45229; 8 Department of Pediatrics, University of Cincinnati, College of Medicine, Cincinnati, OH 45229; 9 Division of Developmental Biology, Cincinnati Children’s Hospital Medical Center, Cincinnati, OH 45229; 10 Department of Ophthalmology, University of Cincinnati, College of Medicine, Cincinnati, OH 45267

**Keywords:** acoustic startle, auditory brainstem response, encephalopsin, light, opsin, panopsin

## Abstract

Opsin-3 (*Opn3*, encephalopsin) was the first nonvisual opsin gene discovered in mammals. Since then, several *Opn3* functions have been described, and in two cases (adipose tissue, smooth muscle) light sensing activity is implicated. In addition to peripheral tissues, *Opn3* is robustly expressed within the central nervous system, for which it derives its name. Despite this expression, no studies have investigated developmental or adult CNS consequences of *Opn3* loss-of-function. Here, the behavioral consequences of mice deficient in *Opn3* were investigated. *Opn3*-deficient mice perform comparably to wild-type mice in measures of motor coordination, socialization, anxiety-like behavior, and various aspects of learning and memory. However, *Opn3*-deficient mice have an attenuated acoustic startle reflex (ASR) relative to littermates. This deficit is not because of changes in hearing sensitivity, although Opn3 was shown to be expressed in auditory and vestibular structures, including cochlear outer hair cells. Interestingly, the ASR was not acutely light-dependent and did not vary between daytime and nighttime trials, despite known functions of *Opn3* in photoreception and circadian gene amplitude. Together, these results demonstrate the first role of *Opn3* on behavior, although the role of this opsin in the CNS remains largely elusive.

## Significance Statement

Despite developmental and adult expression of Opsin-3 (Opn3; encephalopsin) in the cerebral cortex, striatum, thalamus, cerebellum, vestibular and auditory structures, and numerous nuclei of the hypothalamus and brainstem among other areas, mice that lack Opn3 have remarkably normal performance in a variety of cognitive, motor, and auditory tests. This study identifies a role for Opn3 in the potentiation of the acoustic startle reflex (ASR) as the first function of *Opn3* on behavior. While most behaviors assessed do not vary between *Opn3*-expressing and *Opn3*-deficient mice, this study establishes an important baseline into the behavioral physiology of *Opn3* and provides the first insights into the functions of *Opn3* within the central nervous system.

## Introduction

Animals have evolved a family of specialized G protein-coupled receptors, known as opsins, to detect and respond to light. When Opsin-3 (*Opn3*) was discovered, expression was shown in the mammalian brain. The opsin was thus termed encephalopsin (Blackshaw and Snyder, 1999; Halford et al., 2001). More recent analysis has shown that *Opn3* is expressed quite broadly in tissues outside the central nervous system. Several roles for encephalopsin have been defined. These functions include regulation of peripheral clock gene oscillation amplitude (Buhr et al., 2015; Upton et al., 2021) and light-dependent smooth muscle relaxation (Barreto Ortiz et al., 2018; Yim et al., 2019, 2020; Dan et al., 2021; Wu et al., 2021). In addition, *Opn3* in white adipocytes is required for light-dependent enhancement of lipolysis and regulation of body temperature (Nayak et al., 2020; Sato et al., 2020). A role for *Opn3* in skin pigmentation has also been proposed (Haltaufderhyde et al., 2015; Regazzetti et al., 2018; Ozdeslik et al., 2019). In the periphery, functions of *Opn3* were demonstrated to be both light-dependent (Barreto Ortiz et al., 2018; Regazzetti et al., 2018; Yim et al., 2019, 2020; Nayak et al., 2020; Sato et al., 2020; Dan et al., 2021; Wu et al., 2021) and light-independent (Haltaufderhyde et al., 2015; Ozdeslik et al., 2019). There is currently no clear function for *Opn3* within the brain.

*Opn3* is expressed broadly throughout the brain and spinal cord. This expression begins early in neurodevelopment in postmitotic neurons and expression continues throughout life in most brain areas (Olinski et al., 2020; Davies et al., 2021). During development, *Opn3* appears to be expressed in all cranial and spinal nerves, most thalamic nuclei, the cerebellum, cortex, striatum, and several hypothalamic nuclei (Davies et al., 2021). Within the cortex, *Opn3* is expressed in Layer V pyramidal neurons whereas in the striatum, *Opn3* is expressed in *Pthlh*^+^ interneurons (Zeisel et al., 2015, 2018; Muñoz-Manchado et al., 2018). In the cerebellum, *Opn3* is expressed in a subset of Purkinje neurons (Blackshaw and Snyder, 1999; Davies et al., 2021). Given the diverse roles of the various *Opn3*-expressing neurons in the developing and adult mouse, a broad survey of behaviors was required to investigate the role of *Opn3* within the central nervous system.

Across a variety of behaviors, *Opn3*-deficient mouse performance was comparable to wild-type mice except for the acoustic startle reflex (ASR), in which *Opn3*-deficient mice had an attenuated response. Despite previous studies in which *Opn3* functions in circadian rhythmicity and direct light-responsiveness, the ASR was not acutely modulated by time of day or by ambient lighting. Additionally, despite *Opn3* expression in cochlear outer hair cells, loss of *Opn3* did not affect hearing sensitivity or hair cell survival. These results indicated that attenuation of the ASR was likely to be centrally mediated. Remarkably, rotarod performance did not vary by genotype despite high expression of *Opn3* in the striatum, thalamus, and cerebellum. Together, these results identify the first role for *Opn3* on behavior and indicate that the startle response is reduced by the loss of *Opn3*.

## Materials and Methods

### Experimental animals

All animal use was conducted in accordance with protocols approved by the Institutional Animal Care and Use Committee at Cincinnati Children’s Hospital Medical Center. All mice were reared on a 12/12 h light/dark cycle with access to food and water *ad libitum*. Before behavioral experiments, mice were transferred to a 14/10 h light/dark cycle and given at least two weeks to adjust before assessment. For behavior assessment, all mice were two to three months old at the start of experiments. No more than one male and one female per genotype was used per litter to minimize maternal and litter effects. Behavioral testing was conducted in the Animal Behavior Core at Cincinnati Children’s Hospital Medical Center. Experimenters performing behavioral assessments were blinded to genotype. Previously described mouse lines used in this study include: *Opn3-eGFP*, *Opn3^creER^*, *Opn3^lacZ^*, *Opn3^flox^*, *Rosa26^Ai6(ZsGreen)^*, and *Emx1-cre* (Gorski et al., 2002; Buhr et al., 2015; Nayak et al., 2020). Male and female mice were used for all behavioral experiments.

### Behavioral assessments

#### ASR with prepulse inhibition (PPI)

The startle reflex was assessed in SR-LAB apparatus (San Diego Instruments) by placing mice in cylindrical enclosures with a piezoelectric accelerometer attached and within a sound-attenuated chamber as described (Mann et al., 2019). Following a 5-min acclimation period, a 4 × 4 Latin square sequence of trials was presented consisting of: pulse (20 ms, 120 dB SPL white noise with 1.5-ms rise time), pulse with 59 dB SPL prepulse, pulse with 70 dB SPL prepulse, or pulse with 80 dB SPL prepulse. Prepulses were presented for 20 ms and occurred 70 ms before pulse onset. The intertrial interval was between 4 and 12 s. Each set of 16 trials was repeated until a total of 100 trials/d for 2 d. For *Opn3^lacZ^* experiments, mice were tested with the experimental design on two successive days during the circadian day with a white house light on within the sound attenuated chamber. For *Emx1-cre* experiments to assess the role of cortical *Opn3*, light, and time of day, mice were tested on four successive days at either CT2 (subjective day) or CT16 (subjective night) with the house light either on or off and adapted to the light or darkness for 20 min before testing. The response was determined as the maximum amplitude response (mV) within 100 ms of stimulus onset.

#### Auditory brainstem response (ABR)

Three monopolar needle electrodes (a recording at the base of the ear, a reference at the apex of the skull, and a ground at the base of the opposite ear) were placed into a ketamine-xylazine anesthetized mouse. Mice were between two and three months of age. Rectal temperature was monitored via rectal thermometer and controlled via a heating pad for the duration of data collection. Electrodes were connected to a preamplifier [Medusa RA4PA, Tucker Davis Technologies (TDT)] and bandpass filter (0.3–3 kHz) for digitization of the electrophysiologic activity using TDT’s RZ6 processor and BioSigRz software. A speaker (MF1, TDT) was placed 6 cm away from the ear and a microphone (B&K4938, Bruel & Kjaer) was placed immediately next to the left ear for calibration of stimulus intensity. Stimuli were generated with SigGenRz software (TDT) and included 100-μs clicks and 20-ms tonebursts with center frequencies of 8, 16, and 32 kHz with an interstimulus interval of 100 ms. The ABR waveforms were averaged over a minimum of 500 stimulus repetitions. ABR threshold searches were completed by presenting clicks and tonebursts in descending intensity steps of 5 dB from 100 to 10 dB SPL. ABR threshold was identified as the lowest sound intensity in dB SPL at which the waveform peaks (i.e., peaks I–V; see Fig. 2*G*) remained distinguishable from the background electroencephalographic activity.

#### Rotarod performance test

Mice are placed on an automated computer-controlled rotating rod (San Diego Instruments). The speed gradually increased until mice were no longer able to remain on the rod and fell to a padded surface. Total distance traveled and latency to fall were recorded.

#### Three chambered social approach

Mice were placed in the middle chamber of a three-chamber apparatus and allowed to habituate for 10 min then removed. A stranger mouse was placed in a small holding cage in the left or right chamber and the test mouse was placed back in the center chamber and tracked for time spent in each chamber (Amos-Kroohs et al., 2016).

#### Elevated zero maze

Elevated zero maze was performed as described (Mann et al., 2019). The elevated zero apparatus was a 50-cm inner diameter circular platform divided into four quadrants; two opposing quadrants had 15-cm-high walls whereas the remaining two quadrants are open except for a clear 0.5-cm-high acrylic curb. Mice were placed into a walled quadrant and allowed to explore for 5 min. Movement was video recorded and analyzed with AnyMaze software (Stoelting Instruments). The latency to enter an open quadrant, time spent in an open quadrant, and number of entries into an open quadrant were measured.

#### Novel object recognition

Novel object recognition was performed as described (Mann et al., 2019). Mice were habituated to a 40 × 40 cm AnyBox with AnyMaze tracking (Stoelting Instruments) for 10 min on day 1 with a different object in each corner. On the test day, mice were familiarized for 10 min to four identical objects, different from those used in habituation. Next, mice and objects were removed. Following a 1-h delay, mice were returned to the chamber with three copies of the familiar object and one novel object. Mice were given 30 s of combined time to interact with objects. Response was measured as the time interacting with the novel object.

#### Fear conditioning

Fear conditioning took place over 4 d. On day 1 (acquisition), mice were placed into the apparatus (25 × 25 cm; San Diego Instruments) that each had a speaker, light, metal floor grid, and an array of infrared beams (1.5 cm apart in *XY* coordinates) that was situated inside a sound attenuating cabinet. Following a 6-min habituation, mice were conditioned to six tone-light-foot shock pairings. For pairings, the light and tone (85 dB SPL, 2 kHz, 30-s duration) were immediately followed by a foot shock (unconditioned stimulus of 2-s duration, 1.3 mA). Light-tone-shock pairings were separated by 30-s intervals with no stimuli. On day 2 (context-dependent recall), mice were placed back into the apparatus for 6 min with no light-tone. On day 3 (cue-dependent recall), mice were placed in a different apparatus (varying in shape, color, and texture). For the first 3 min, no stimuli or shock was presented followed by 10 trials of alternating 30 s periods of stimulus-on and 30 s of stimulus-off, but no foot shock. On day 4 (extinction), mice were again placed in the apparatus used on day 3 and presented with five trials of alternating stimulus-on and stimulus-off trials. Number of beam breaks were analyzed.

#### Morris water maze

Morris water maze tests was performed as described (Vorhees and Williams, 2006; Mann et al., 2019). For all trials, mice were placed in a white polyethylene circular pool (122-cm diameter, 51 cm deep) filled to a depth of 25 cm with room temperature water (∼21°C). Distinctive visual cues were placed on the walls outside the pool. Mice were tested in four phases: training, acquisition, reversal, and cued (Vorhees and Williams, 2006). When present, the platform was placed 1.5 cm under the surface of the water and was white as was the tank. Training consisted of five to six trials on 1 d with a visible platform from a fixed start to a fixed goal to expose mice to the test conditions. Acquisition (platform in the SW quadrant) and reversal (platform in the NE quadrant) consisted of four trials (90 s maximum per trial)/d for 5 d to a hidden platform from pseudo-random start positions and the platform in a fixed location and a probe trial (45 s) before platform trials on day 3 and a second probe trial on day 6 with no platform. Cued consisted of four trials/day for 2 d with a visible platform and curtains closed around the pool to obscure distal cues with start and platform positions randomized on each trial to prevent spatial navigation as a test of proximal cue learning. Tests were video recorded and analyzed with AnyMaze software (Stoelting Instruments).

### Immunofluorescence staining and confocal imaging

Unless otherwise specified, all mice used in imaging experiments were anesthetized with isoflurane and transcardially perfused with PBS followed by 4% paraformaldehyde (PFA) in PBS. Brains were postfixed in PFA overnight at 4°C and then cryoprotected in 15% and 30% sucrose. Brains were embedded and sectioned with a cryostat (Leica CM3050 S). For inner ear samples, perfusion fixed samples were dissected and mounted on glass slides. For immunofluorescence, sections were incubated in primary antibody. After primary staining, sections were washed and stained with fluorescent secondary antibodies. Finally, sections were counterstained with either Hoechst 33 342 (1:1000; Invitrogen) or Nissl (1:100; NeuroTrace 435/455, Invitrogen) or phalloidin 594 (1:100; ATT Bioquest) before cover slipping. All images were captured with a Zeiss LSM700 confocal microscope.

### Quantification and statistical analysis

Behavioral data were analyzed using generalized linear mixed-effect models with repeated-measures (SAS version 9.2, SAS Institute). Results were considered statistically significant if *p* < 0.05. Variance-covariance matrices of best fit were used with Kenward–Roger first order estimated degrees of freedom. Follow-up analyses were conducted by slice-effect ANOVAs for significant interactions. For ABR experiments, two-way ANOVA was performed across genotypes and frequencies for narrowband stimuli and a two-way unpaired *t* test was performed for click-evoked responses. Summary and statistical table for each test can be found in Extended Data Table 1-1.

## Results

### *Opn3* potentiates the ASR

**Figure 1.**
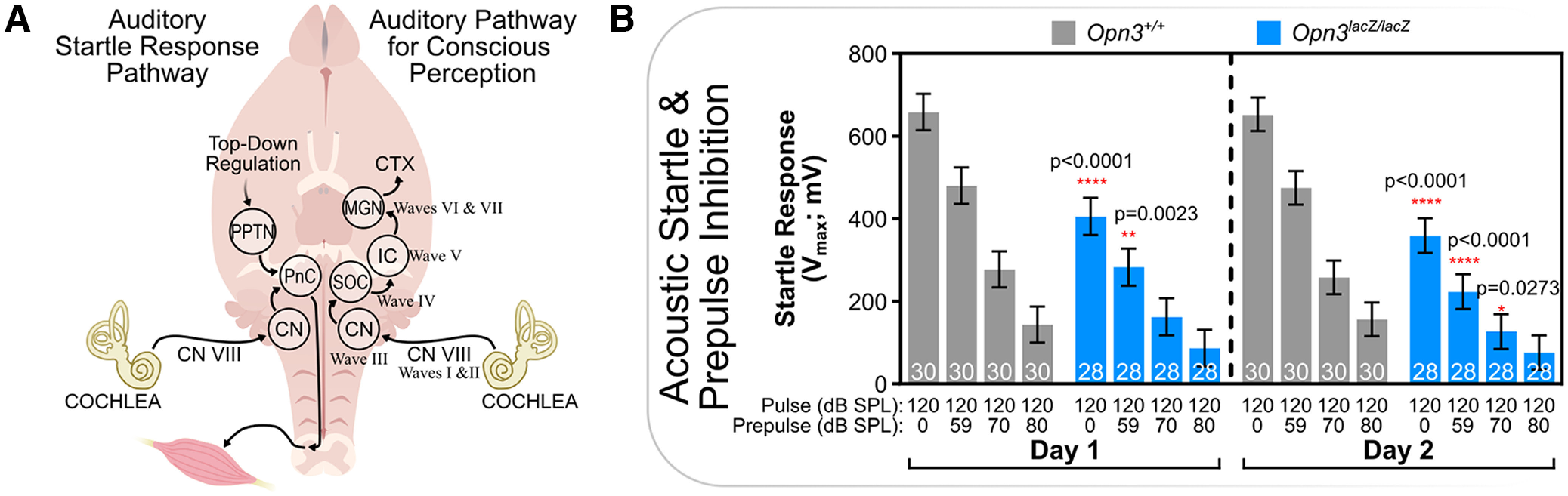
ASR, but not PPI, is potentiated by *Opn3*. ***A***, Left, Pathway from hair cells within the cochlea to muscle twitch in the ASR. Right, Pathway from hair cells within the cochlea to cortex for conscious auditory perception and anatomic location of waves generated during the ABR. ***B***, Pulse (120 dB SPL) was presented alone or with 59, 70, or 80 dB SPL prepulse. Same protocol was used across days. *P*-values reflect significant effects of genotype at given prepulse intensity for each given day. CN, cochlear nucleus; CN VIII, vestibulocochlear nerve; CTX, cortex; IC, inferior colliculus; MGN, median geniculate nucleus; PnC, pontine reticular nucleus; PPTN, pedunculopontine tegmental nucleus; SOC, superior olivary complex. See Extended Data Figure 1-1 for additional data. Data represent mean+/− s.e.m.

When a loud noise occurs, the sound is detected by hair cells in the cochlea, which transmit this information via the vestibulocochlear nerve (CN VIII) to the cochlear nucleus (Fig. 1*A*). For startle-eliciting noises, the cochlear nucleus excites giant cells of the caudal pontine reticular nucleus (PnC), which synapse directly onto lower motor neurons within the spinal cord to generate a flinch (Koch et al., 1993; Fendt and Koch, 1999; Fendt et al., 2001; Gómez-Nieto et al., 2014a, b). This pathway occurs independent of conscious perception and is distinct from the pathway involved in auditory perception (Fig. 1*A*). Neurons of the PnC are primarily regulated by inhibitory acetylcholine release from the pedunculopontine tegmental nucleus (PPTN), both of which express *Opn3* during development (Davies et al., 2021). In turn, the PPTN is part of a larger, higher-order circuit involved in top-down regulation (Gut and Winn, 2016). One way in which the ASR can be modified is by presentation of stimuli (prepulse) immediately preceding the startle stimulus (pulse). A prepulse stimulus can be auditory, visual, or tactile (Yeomans et al., 2002). When a prepulse is presented during particular intervals before the pulse, the startle reflex is attenuated, a phenomenon known as PPI (Plappert et al., 2004). This complex neurologic process is known as sensorimotor gating and is involved in the filtering of salient stimuli from irrelevant stimuli (the cocktail party effect; Swerdlow et al., 2016). Sensorimotor gating is influenced by sensory systems, attentional processing, motivational salience, and arousal state. Interestingly, PPI is known to be impaired in individuals with season-of-birth-dependent neurologic disorders such as schizophrenia and autism spectrum disorders as well as their first-degree relatives (Braff et al., 2001; Greenwood et al., 2007, 2013; Perry et al., 2007; Thaker, 2008; Beck and Catuzzi, 2013; Kohl et al., 2013, 2014; Seidman et al., 2015; Takahashi et al., 2016).

10.1523/ENEURO.0202-22.2022.tab1-1 Extended Data Table 1-1 Statistical table. It contains relevant statistical information for all statistical tests performed, included test performed for each experiment, *n* for each condition, and all *p*-values. Download Table 1-1, XLS file.

10.1523/ENEURO.0202-22.2022.f1-1 Extended Data Figure 1-1 ASR and PPI is not acutely dependent on lighting or time of day, but is inhibited by cortical *Opn3*. Average startle response in *Opn3* wild-type mice at either 2 h after lights-on or 2 h after lights-off in darkness or under light exposure. ***A***, Averages for both combined males and females, or (***B***) males and (***C***) females analyzed separately. Download Figure 1-1, TIF file.

PPI is mediated via a circuit of brain regions including the striatum, cortex, cerebellum, and thalamus, all of which express *Opn3* (Blackshaw and Snyder, 1999; Swerdlow et al., 2016; Gómez-Nieto et al., 2020; Olinski et al., 2020; Davies et al., 2021). For these reasons, ASR with and without PPI was assessed in *Opn3^+/+^* and *Opn3^lacZ/lacZ^* mice. Interestingly, while PPI was intact in *Opn3*-deficient mice, the baseline ASR was attenuated (Fig. 1*B*). As *Opn3* expression can be detected throughout multiple components of the circuit regulating ASR, these results may either be the result of impaired startle detection (i.e., poor hearing), increased inhibition of PnC giant cells from the PPTN, or an impaired motor response.

**Figure 2.**
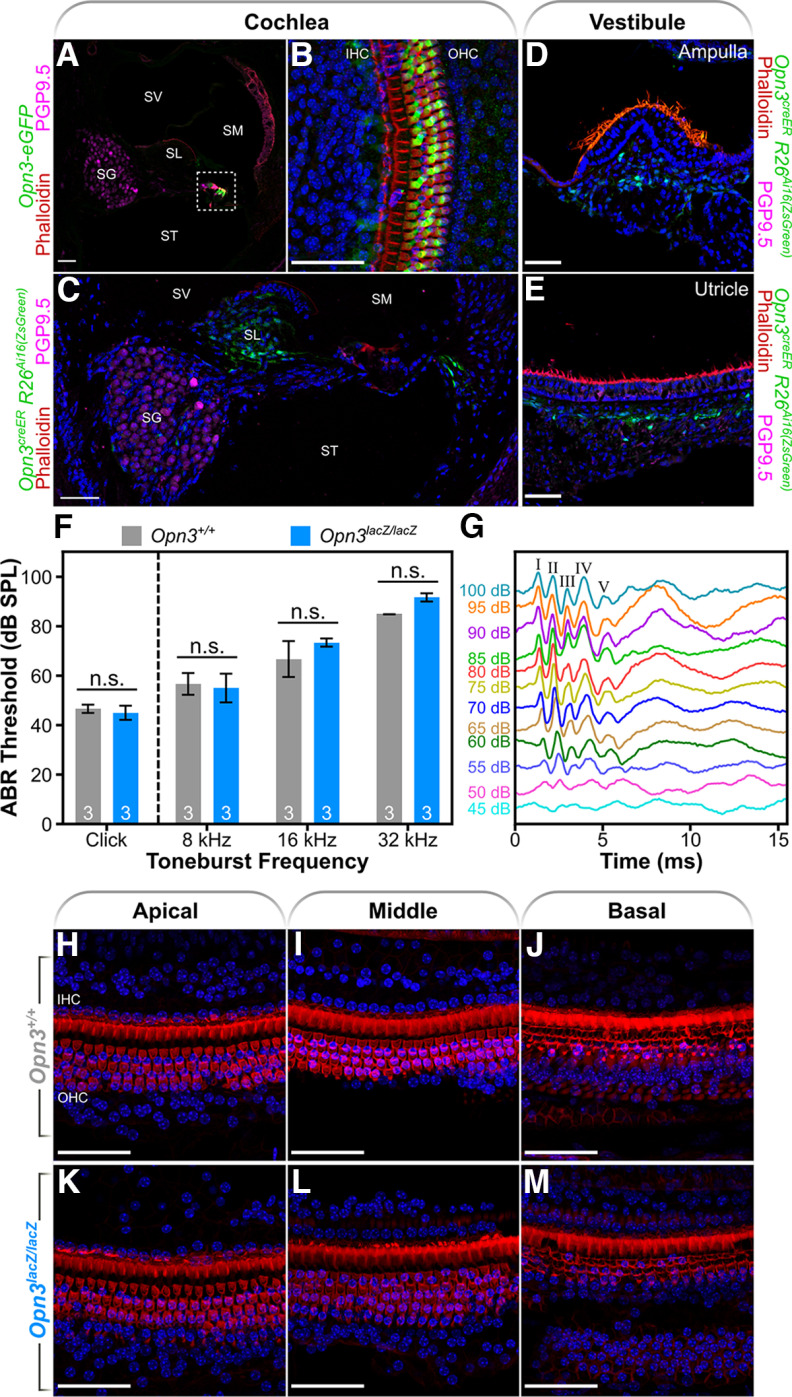
*Opn3* and the auditory system. ***A***, ***B***, Expression of *Opn3-eGFP* in outer hair cells of the cochlea and (***C***) expression of *Opn3^creER^*-dependent reporter in cochlear and (***D***, ***E***) vestibular structures. ***F***, ABRs in *Opn3^+/+^* and *Opn3^lacZ/lacZ^* mice to click and low, medium, and high frequencies. ***G***, Representative ABR with five peaks indicated at top. ***H–M***, Representative cochlear hair cells from three-month-old *Opn3^+/+^* and *Opn3^lacZ/lacZ^* mice. IHC, inner hair cell; OHC, outer hair cells; PGP9.5, protein gene product 9.5; SG, spiral ganglion; SL, spiral limbus; SM, scala media; ST, scala tympani; SV, scala vestibuli. Scale bar: 50 μm. n.s., Not Significant. Data represent mean +/− s.e.m.

**Figure 3.**
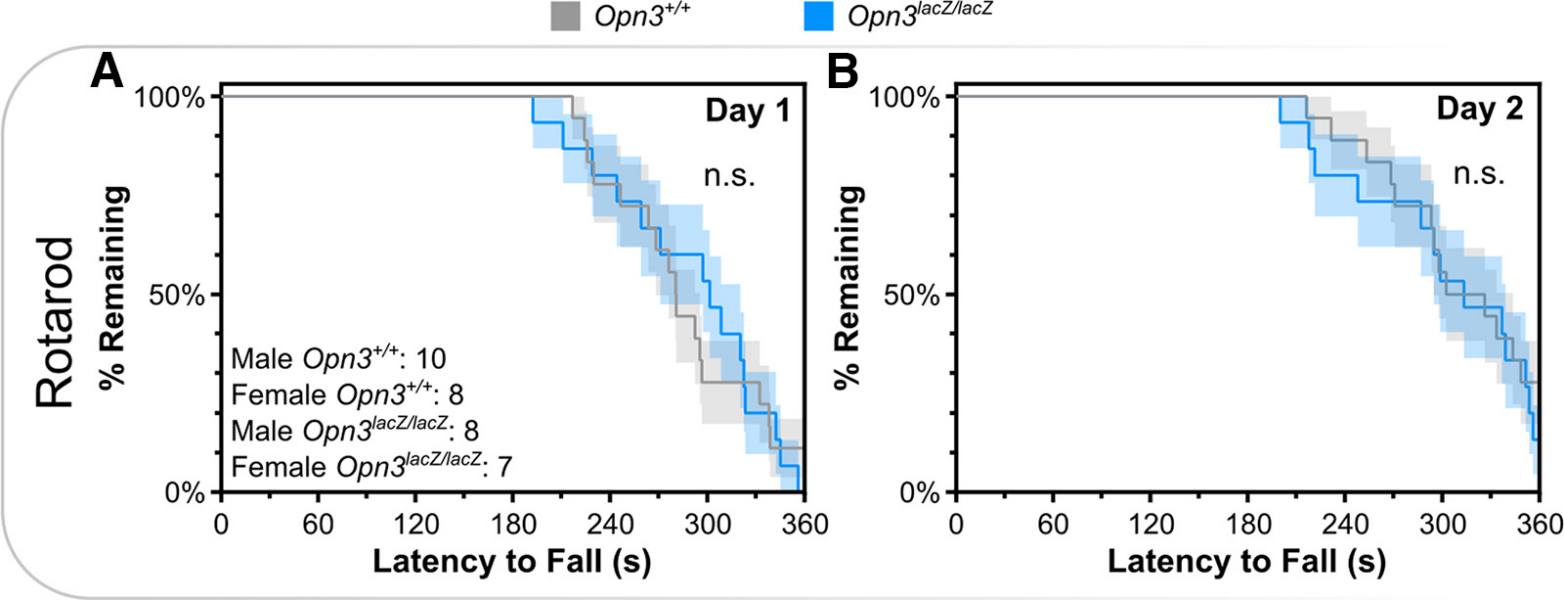
*Opn3*-deficient mice have normal motor performance. ***A***, ***B***, Kaplan–Meier curves of 2-d rotarod performance. n.s., Not Significant. Data represent mean +/− s.e.m.

The auditory system was investigated first. *Opn3* expression was detected in numerous vestibular and auditory structures, including the cochlear outer hair cells, spiral limbus, spiral ganglion and root cells of the cochlea, cristae ampullaris, and utricular macula using two distinct reporter mouse lines (Fig. 2*A–E*). Despite this expression, *Opn3*-deficient mice had normal hearing sensitivity to a range of acoustic stimuli, including broadband and narrowband sounds (Fig. 2*F*,*G*). Additionally, there was no evidence of worsened age-related hair cell loss in *Opn3*-deficient mice (Fig. 2*H–M*). Taken together, no differences in hearing sensitivity or hair cell survival were found that could explain the attenuated ASR. Additionally, there were no deficits in motor performance in *Opn3*-deficient mice that would suggest that *Opn3*-deficient mice would have an impaired ability to generate a normal muscle contraction (see below; Fig. 3; Extended Data Fig. 8-1*C*,*F*,*I*,*L*). Thus, by elimination, the attenuated ASR appears to be mediated via increased central inhibition of the reflex.

The ASR and PPI were repeated with a protocol in which mice were either light-adapted or dark-adapted before test trials to assess whether the startle reflex could be acutely modulated by light. Additionally, experiments took place either 2 h after lights-on (CT2) or 2 h after lights-off (CT16) to assess whether there was a circadian influence on the response. Mice with conditional loss of *Opn3* from the cortex were used, as this was hypothesized to be a potential source of *Opn3*-dependent top-down modulation of the ASR. Cortical deletion of Opn3 was achieved using *Emx1-cre*, which is expressed in excitatory neurons of the cortex and hippocampus, including cortical *Opn3*-expressing pyramidal neurons but not in other *Opn3*-expressing domains (Gorski et al., 2002). The results indicate that ASR is not acutely modulated by light (Extended Data Fig. 1-1). This indicates that the previous finding that the acoustic startle is attenuated in *Opn3*-deficient mice is not because of an acute potentiation by light acting via Opn3. Additionally, the ASR and PPI did not vary at two different times of day, during the active and inactive phases (Extended Data Fig. 1-1). This result suggests that the mechanism by which *Opn3* facilitates the ASR is not dependent on *Opn3*-dependent clock gene amplitude, as seen in other non-neural *Opn3*-expressing tissue (Buhr et al., 2015; Upton et al., 2021). Surprisingly, loss of cortical *Opn3* resulted in a potentiation of the ASR, rather than an attenuation as originally observed in germline knock-out mice (Extended Data Fig. 1-1). These findings also demonstrated a sexual dimorphism that was not previously observed in *Opn3^lacZ^* mice, in which the entirety of the effect of genotype was driven by a potentiated response in males (Extended Data Fig. 1-1). Taken together, these results indicate that *Opn3* modulates the ASR, although not via acute light sensitivity or circadian regulation. Alternative mechanisms by which *Opn3* could regulate ASR include developmental effects of the loss of *Opn3*, chronic light exposure, or through light-independent mechanisms. The attenuation seen in *Opn3^lacZ/lacZ^* mice does not appear to be via cortical expression of *Opn3*, although cortical *Opn3* may function in conjunction with other *Opn3*-expressing domains to modulate the response.

### Additional behavioral assessments

**Figure 4.**
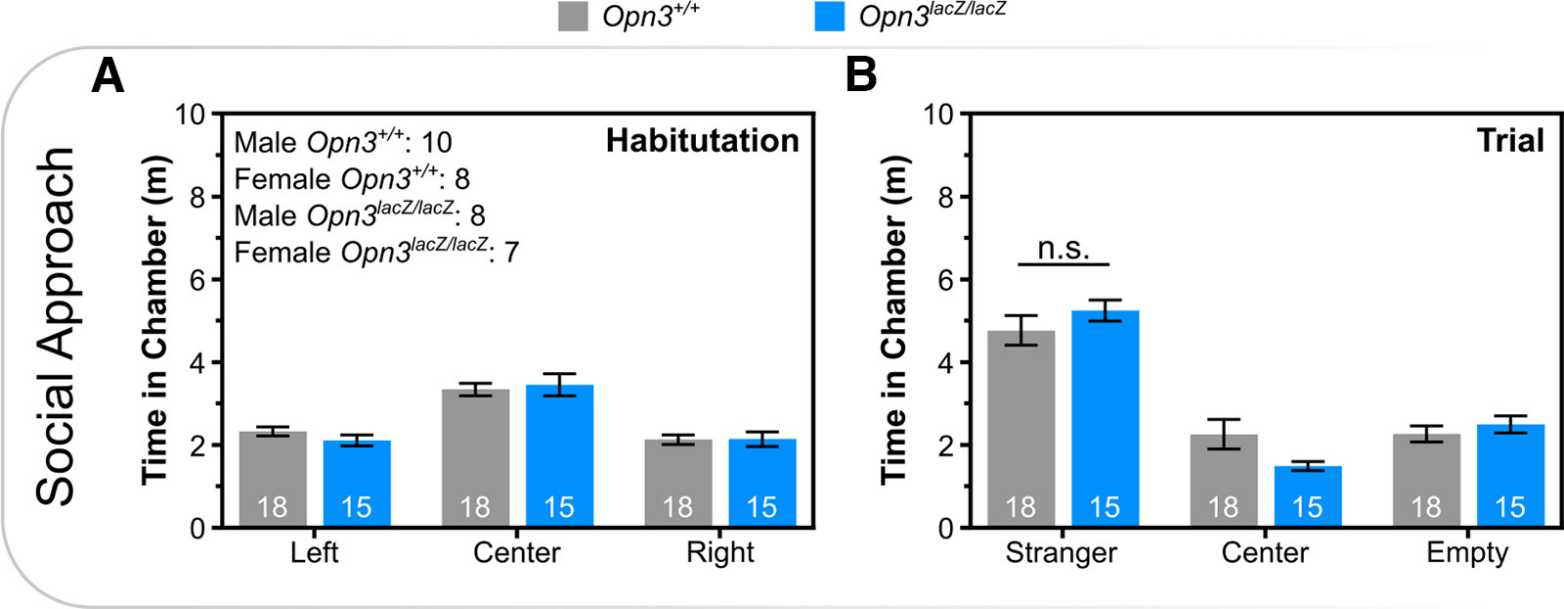
*Opn3*-deficient mice have normal sociability. Time spent in chamber during (***A***) habituation and (***B***) when a stranger was placed in a cage within one chamber during three-chambered social approach. Data represent mean +/− s.e.m.

**Figure 5.**
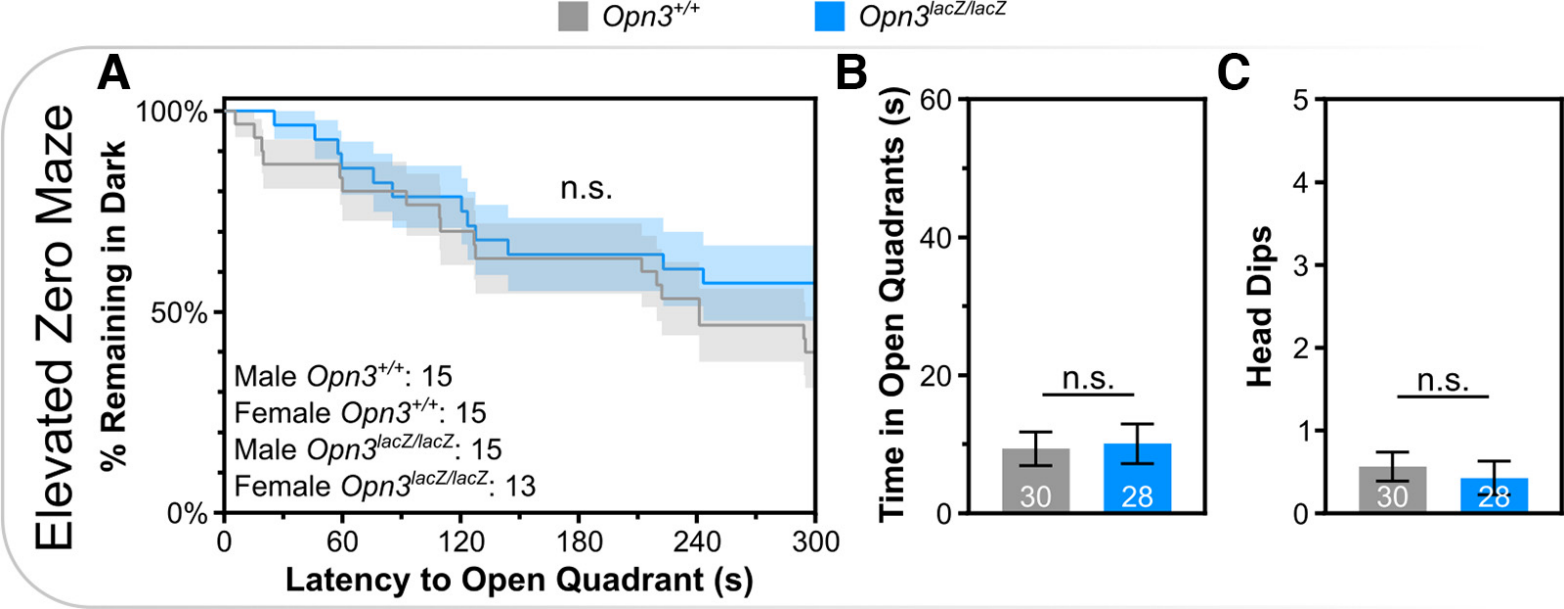
Opn3-deficient mice do not demonstrate anxiety-like behavior. ***A***, Kaplan–Meier curve of latency to enter an open quadrant during elevated zero maze. ***B***, Total time spent in open quadrants and (***C***) number of head dips during elevated zero maze. n.s., Not Significant. Data represent mean +/− s.e.m.

Based on the broad expression of *Opn3*, other behaviors were assessed. Although *Opn3* is expressed in Purkinje neurons of the cerebellum, *Pthlh^+^* interneurons of the striatum, and vestibular structures in the inner ear, which function in coordination, motor function, and balance, respectively, there were no deficits in rotarod performance (Fig. 3). Despite expression in Layer V pyramidal neurons and oxytocinergic neurons of the PVN, which function in social behavior, there were no *Opn3*-dependent differences on the three-chambered social preference test (Fig. 4). Similarly, there was no difference in anxiety-like behavior, as assessed via latency to enter open quadrants, time spent in open quadrants, or head dips during elevated zero maze (Fig. 5).

**Figure 6.**
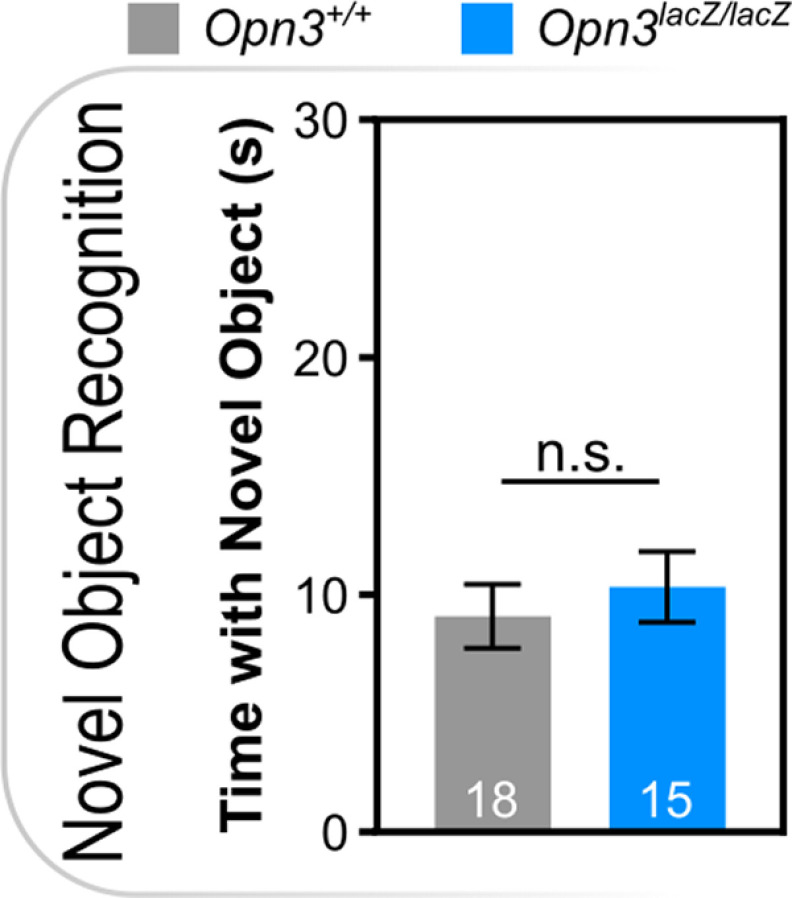
Novel object recognition is independent of *Opn3*. n.s., Not Significant. Data represent mean +/− s.e.m.

**Figure 7.**
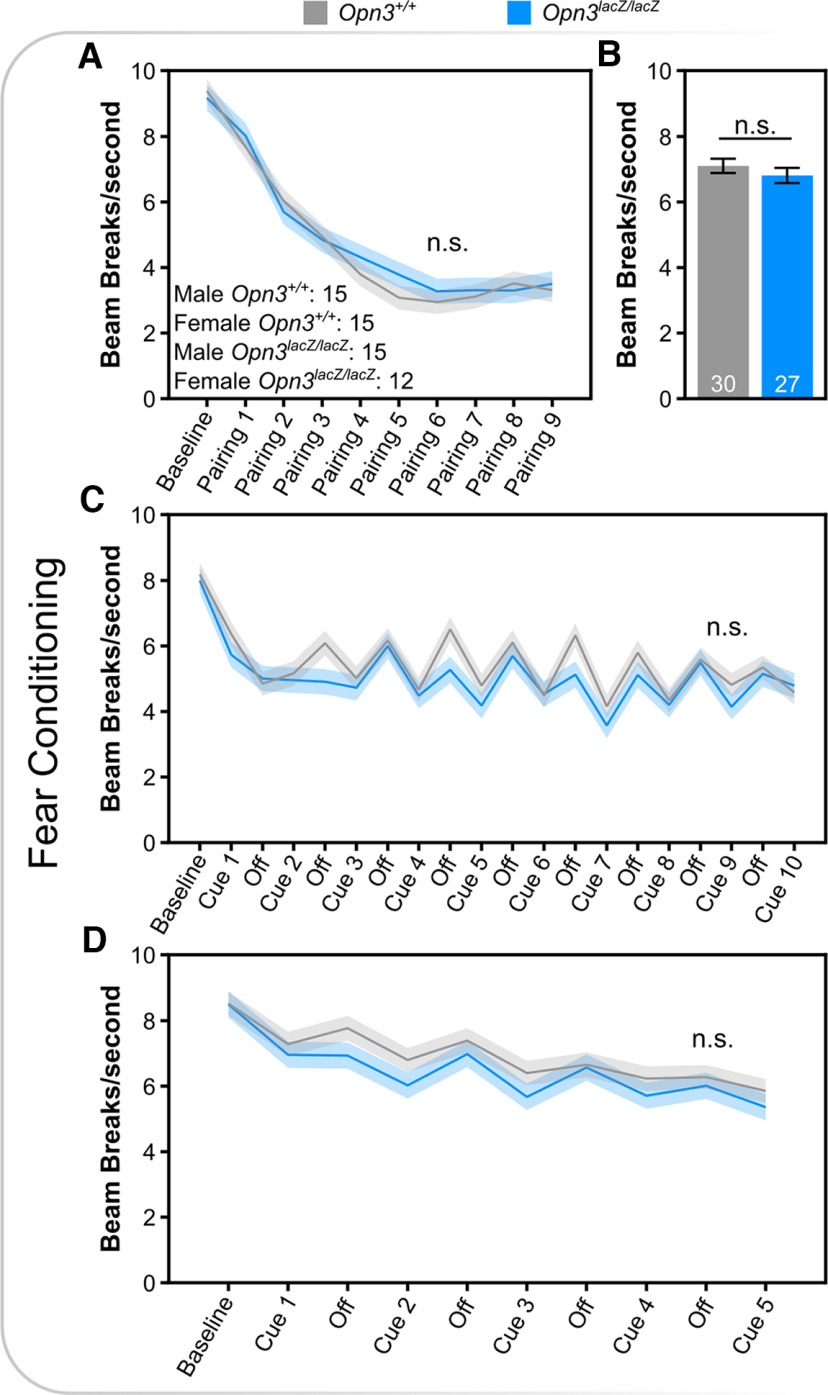
Fear conditioning is not dependent on Opn3. ***A***, Locomotor activity during conditioning between foot-shock (unconditioned stimulus) and an auditory tone (conditioned stimulus). ***B***, Locomotor activity during context-dependent recall. ***C***, Locomotor activity during cue-dependent recall in which the conditioned stimulus is re-presented in the absence of the unconditioned stimulus. ***D***, Extinction of fear conditioning. n.s., Not Significant. Data represent mean +/− s.e.m.

**Figure 8.**
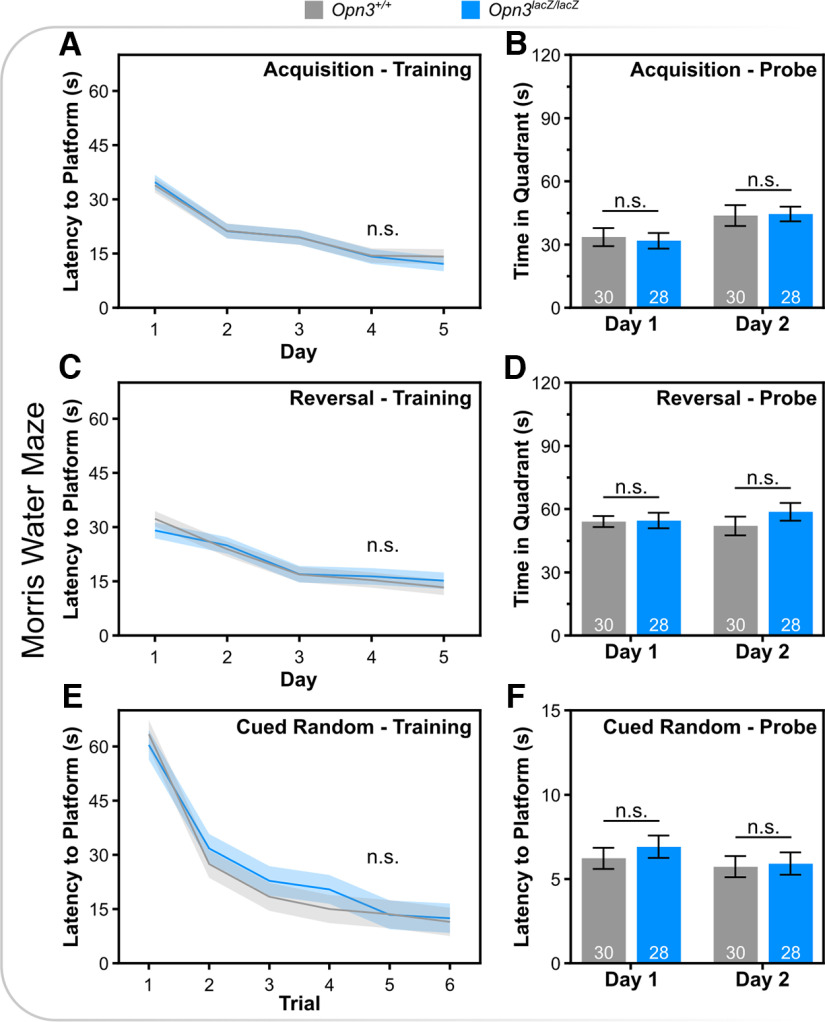
Morris water maze performance in wild-type and *Opn3*-deficient mice. ***A***, Latency to submerged platform averaged across daily trials for five subsequent days during initial spatial learning. ***B***, Time spent in target quadrant once submerged platform was removed on two subsequent days. ***C***, Latency to submerged platform averaged across daily trials for five subsequent days during reversal phase. ***D***, Time spent in target quadrant once submerged platform has been removed following spatial learning when platform was in reversal position. ***E***, Latency to submerged platform during cued-random phase of training and (***F***) latency to the cued platform when probed on subsequent days. See Extended Data Figure 8-1 for additional data. n.s., Not Significant. Data represent mean +/− s.e.m.

Given the expression in brain areas associated with higher order functions (i.e., cortex, striatum, cerebellum, thalamus), various types of memory were assessed including hippocampal-dependent memory, amygdala-dependent memory, and spatial memory. *Opn3*-deficient mice did not have differences in novel object recognition, a test of hippocampal-dependent incidental memory (Fig. 6). Similarly, *Opn3*-deficient mice did not show differences in context-recall, cued-recall, or extinction in fear conditioning (Fig. 7). Lastly, in the Morris water maze, a measure of spatial learning and cognitive flexibility, *Opn3*-deficient mice did not show differences in acquisition or reversal, and no differences in proximal cue learning in the visible platform phase of testing (Fig. 8). Furthermore, there were no differences in path length or efficiency and *Opn3*-deficient mice had similar swim speeds as control mice, again reflecting normal motor performance (Extended Data Fig. 8-1). Similar to ASR data in *Opn3^lacZ/lacZ^* mice, none of the above behavioral tests demonstrated an interaction between genotype and sex (Extended Data Table 1-1).

10.1523/ENEURO.0202-22.2022.f8-1 Extended Data Figure 8-1 Morris water maze performance is independent of *Opn3*. Path length to platform (***A***) and path efficiency per day (***B***) averaged across trials during initial acquisition of spatial memory. ***C***, Mean swim speed per day averaged across trials during initial acquisition of spatial memory. Distance (***D***), platform crossings (***E***), and average swim speed (***F***) when hidden platform was removed for two subsequent days. Path length to platform (***G***) and path efficiency per day (***H***) averaged across trials during reversal learning. ***I***, Mean swim speed per day averaged across trials during reversal of platform location. Distance (***J***), platform crossings (***K***), and average swim speed (***L***) when hidden platform was removed for two subsequent days. Download Figure 8-1, TIF file.

## Discussion

Despite widespread expression of *Opn3*, mice that lack *Opn3* are remarkably similar to wild-type littermates with only subtle behavioral phenotype differences. Perhaps the most surprising result is that *Opn3*-deficient mice have normal motor performance, both as assessed via rotarod performance as well as swim speed during the Morris water maze. This normal performance is despite loss of *Opn3* from vestibular structures in the inner ear, striatal interneurons, thalamic nuclei, and the cerebellum. All these structures in different ways contribute to balance and motor coordination. Also surprising was normal hearing sensitivity across the auditory spectrum despite loss of *Opn3* from outer hair cells, and normal social approach, despite loss of *Opn3* from oxytocinergic paraventricular neurons. One pattern that has emerged from various studies on *Opn3* is that there are seldom baseline phenotypes. For example, *Opn3*-deficient mice have a normal baseline body temperature at room temperature. However following cold exposure, *Opn3*-deficient mice are unable to maintain their body temperature when compared with control animals (Nayak et al., 2020). Similarly, with *ad libitum* access to standard chow, *Opn3*-deficient mice have normal body mass, however in response to high fat diet, *Opn3*-deficient mice gain more weight and have increased adiposity than wild-type controls (Nayak et al., 2020; Sato et al., 2020). Thus, the lack of a phenotype in many of these behavioral paradigms may reflect a lack of *Opn3* function in baseline behavior, however, under particular stressors, an *Opn3*-dependent phenotype may manifest. In this manner, *Opn3* may function in a context-dependent manner across the various tissues in which it is expressed.

Of the behaviors assessed, only the ASR had a clear *Opn3*-dependent effect on performance. In the absence of germline *Opn3*, these mice had attenuated startle reflexes. These results were consistent with a role for *Opn3* in the potentiation of the reflex, via increased top-down facilitation, or in the disinhibition of the reflex, via decreased top-down suppression of this reflex. Interestingly, this effect was not acutely modulated by light or time of day, suggesting three possible explanations. First, that *Opn3* may alter neurodevelopment given its robust expression early in development, although no developmental effects of *Opn3* have been noted to date (Davies et al., 2021). Second, *Opn3* may function in the CNS on longer timescales than 20 min. However, all light-dependent effects of *Opn3* have been demonstrated on the order of seconds to minutes, so this explanation seems unlikely given that it would contradict previous findings (Barreto Ortiz et al., 2018; Yim et al., 2019; Nayak et al., 2020; Sato et al., 2020; Wu et al., 2021). Lastly, *Opn3* may regulate this pathway independent of light. as has been observed in other peripheral tissue (Ozdeslik et al., 2019). The findings that the ASR did not vary between the active and inactive phases is in contrast to ASR findings in rats, in which the response is greater during the active phase (Chabot and Taylor, 1992; Frankland and Ralph, 1995; Longenecker et al., 2018). Study design differences should be noted, including that this study involves mice rather than rats and that this study included PPI throughout ASR trials.

An interesting pattern that is emerging in *Opn3* expression is that it is often found in multiple regions of the same pathway. For example, in the osmoregulatory system, *Opn3* is expressed in the subfornical organ, the median preoptic nucleus, the paraventricular nucleus, and the supraoptic nucleus (Davies et al., 2021). Similarly, within the visual system, *Opn3* is detected in RGCs in the retina, the LGN in the thalamus, and the visual cortex (Davies et al., 2021). Likewise, *Opn3* is detected in hair cells of the cochlea, medial geniculate nucleus of the thalamus, and the auditory cortex (Davies et al., 2021). This pattern continues for more complex sensory systems, such as balance/coordination where *Opn3* is detected in the ampulla and utricle as well as the cerebellum, striatum, and motor cortex. This complex pattern of expression may underlie its function in these circuits and may be responsible for the findings in ASR experiments.

Surprisingly, the conditional deletion of *Opn3* from the cortex resulted in a potentiation of the ASR in males only. Not only was the direction of effect opposite than that seen in germline *Opn3*-deficient animals, but there was no difference between sexes in the germline knock-out. One explanation for this observation is that multiple *Opn3*-expressing domains are contributing to the regulation of the startle reflex in an *Opn3*-dependent manner. In the cortex, *Opn3* would appear to attenuate the reflex, whereas in another brain area, *Opn3* might function to potentiate the reflex. The germline results may therefore reflect either a summation of these responses, if the nuclei function in parallel, or only reflect this noncortical contribution, if the nuclei function in series. This effect could be investigated in future studies using various mouse lines to target additional and overlapping *Opn3*-expressing domains. For example, a pan-neuronal cre-line would more directly support a central role for *Opn3* in regulating the ASR. Telencephalon-specific cre-lines (e.g., *Foxg1-cre*) and striatum-specific cre lines (e.g., *Dlx1/2-cre*) could be compared with those that are cortex-specific (*Emx1-cre*) to better understand the role of *Opn3* in top-down modulation of this reflex from various regions of the brain. Interestingly, all three types of estrogen receptors (α, β, and G protein coupled estrogen receptor 1) are expressed in the cortex, which may provide a source of interaction between *Opn3* and the sex of the mouse (Zeisel et al., 2015, 2018; Ye et al., 2019; Zang et al., 2020). It is worth summarizing that this is the only condition in which an *Opn3*-dependent phenotype demonstrates differences between males and females, both within this study of behavior and previous studies, although both sexes have not been routinely examined (Nayak et al., 2020; Sato et al., 2020; Dan et al., 2021; Wu et al., 2021). This result further reiterates the need to investigate both male and female animals, unless there is a biological or mechanistic reason they should be analyzed separately (Yim et al., 2020). Further work will be needed to identify the source of this reversal of effect on the ASR and interaction with sex.

Overall, these results and the ubiquity of *Opn3* expression, particularly in development, suggest that *Opn3* may have important roles in various neurologic systems. During development, *Opn3* is the first opsin expressed, around embryonic day 9.5, and during neurodevelopment, is expressed in every spinal nerve and cranial nerve as well as a variety of cortical and subcortical structures (Davies et al., 2021). The variety of cells expressing *Opn3* likely suggests that its role in these neurons is more global, rather than specific to a particular neural/sensory system. Despite this widespread expression of *Opn3*, their behavior is remarkably normal. There may be functional redundancy between Opn3 and another protein which would hinder changes in the *Opn3* loss-of-function mice. Thus, while *Opn3* likely has developmental functions within the nervous system given its breadth of expression, its precise role remains to be determined.

This study represents a broad assessment of *Opn3* under baseline conditions. Most behaviors were unaffected by germline loss of *Opn3*, including locomotor and auditory functions, with the exception of the ASR. Given the lack of baseline effects in other *Opn3*-dependent functions, elucidating the role of centrally expressed *Opn3* may require additional stressors or else, loss of *Opn3* may mediate susceptibility to other neurologic insult, either developmentally or in the adult. Based on previous studies on *Opn3*, these results would predict that conditions that increase hypervigilance in an animal would exacerbate the difference in ASR between mice with and without *Opn3*.

## Synthesis

Reviewing Editor: David Schoppik, New York University - Langone Medical Center Decisions are customarily a result of the Reviewing Editor and the peer reviewers coming together and discussing their recommendations until a consensus is reached. When revisions are invited, a fact-based synthesis statement explaining their decision and outlining what is needed to prepare a revision will be listed below. The following reviewer(s) agreed to reveal their identity: Ana Maria de Lauro Castrucci.

1. Please check the references; several were incomplete.

2. One of the reviewers felt that it would be worth highlighting the phenotype that was discovered (ASR). Their suggestion was to adjust the title to reflect the finding, and to move the panel of behavioral experiments to supplemental. This point did not reflect consensus, as others felt that given eNeuro’s call for “negative” data it was just as reasonable to focus on the complete set of tests performed. I therefore mention this as a suggestion for your consideration.

3. The logic of lumping motor, social and anxiety-like behaviors in Fig 3 and then having fear conditioning, novel objection recognition, and MWM in subsequent figures is not clear. If there was a rationale behind this particular choice of organization please make it clear.

4. It is mentioned in the discussion that OPN3 may function in a context-dependent manner -- Would it be possible to introduce a stressor for your ASR tests? Could this increase the effect size of the phenotype you observe? The reviewers felt that this deserved consideration in the Discussion.

5. The potentiation of cortical OPN3 loss is confusing and not explained fully. Since it is hypothesized that the attenuated ASR is CNS controlled, could you use a brain-wide cre (nestin-cre?). We agreed that it would be possible to deal with this in the Discussion, rather than repeating experiments with another line of mice.

6. In addition, the sexually dimorphic phenotype was only mentioned in passing but I feel could be a point of further investigation. Did you observe sexually dimorphic phenotypes in OPN3 deficient mice?

7. Would it be clearer to put the bars side-by-side rather than split by genotype in the Extended Data to facilitate identification of the significant conditions? It’s a bit challenging to follow the logic.
