# Supplementary figures and images for: Comprehensive Behavioral Analysis of Opsin 3 (Encephalopsin)-Deficient Mice Identifies Role in Modulation of Acoustic Startle Reflex
Source: eNeuro. 2022 Sep 27;9(5):ENEURO.0202-22.2022. doi: 10.1523/ENEURO.0202-22.2022 (PMC9532019; doi:10.1523/ENEURO.0202-22.2022)

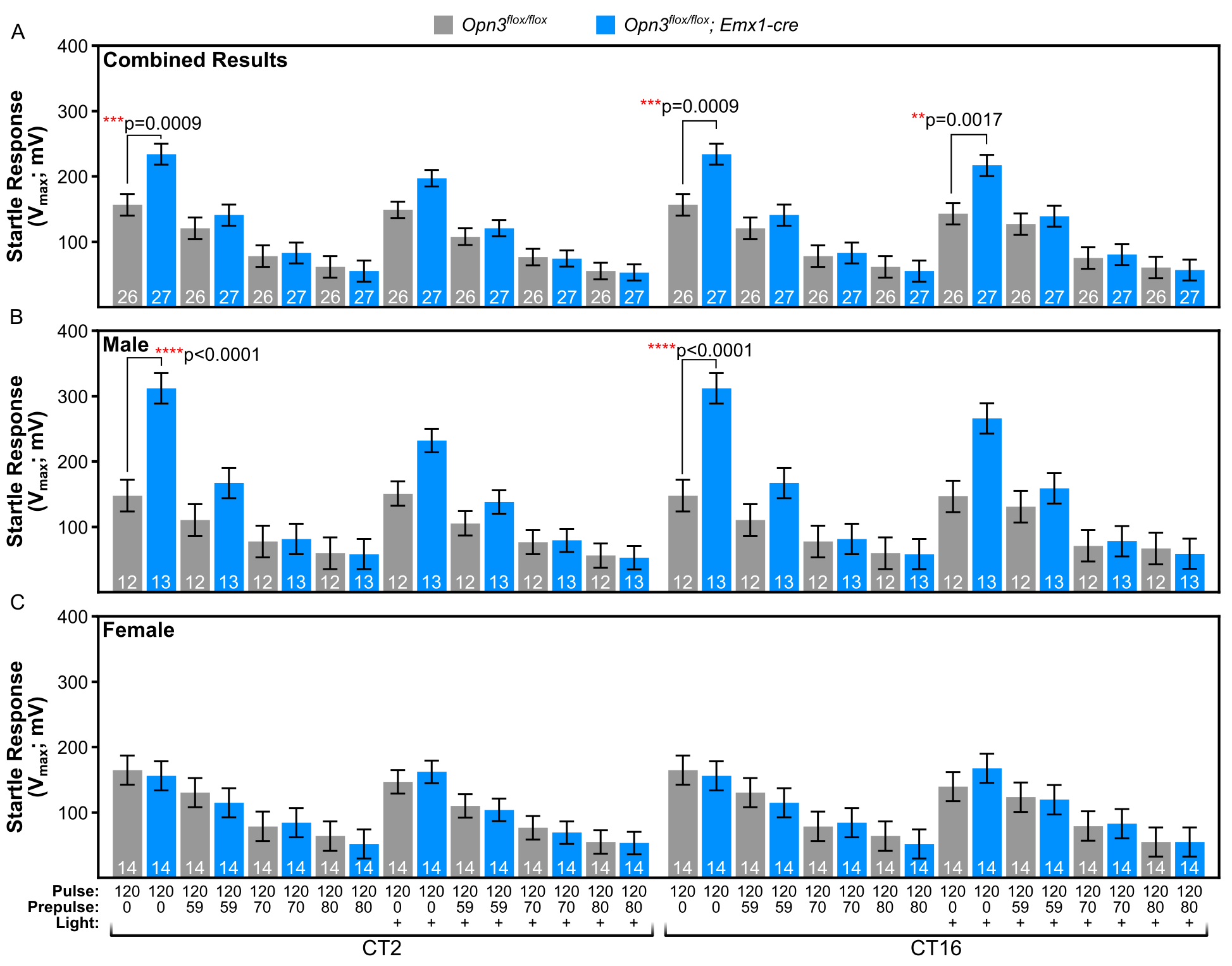

Supplement: Extended Data Figure 1-1 — ASR and PPI is not acutely dependent on lighting or time of day, but is inhibited by cortical Opn3. Average startle response in Opn3 wild-type mice at either 2 h after lights-on or 2 h after lights-off in darkness or under light exposure. A, Averages for both combined males and females, or (B) males and (C) females analyzed separately. Download Figure 1-1, TIF file. [file enu-eN-NWR-0202-22-s02.tif]

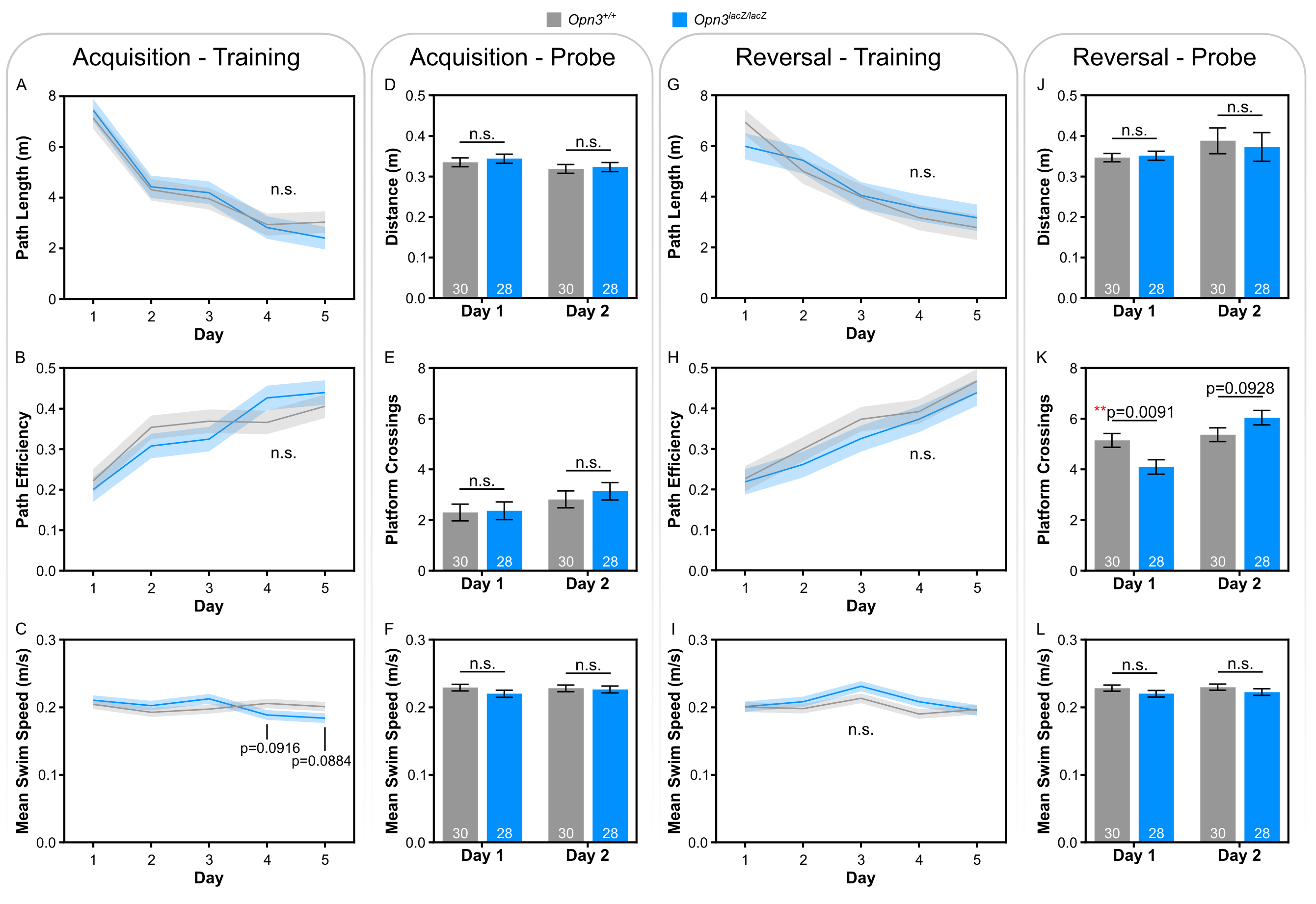

Supplement: Extended Data Figure 8-1 — Morris water maze performance is independent of Opn3. Path length to platform (A) and path efficiency per day (B) averaged across trials during initial acquisition of spatial memory. C, Mean swim speed per day averaged across trials during initial acquisition of spatial memory. Distance (D), platform crossings (E), and average swim speed (F) when hidden platform was removed for two subsequent days. Path length to platform (G) and path efficiency per day (H) averaged across trials during reversal learning. I, Mean swim speed per day averaged across trials during reversal of platform location. Distance (J), platform crossings (K), and average swim speed (L) when hidden platform was removed for two subsequent days. Download Figure 8-1, TIF file. [file enu-eN-NWR-0202-22-s03.tif]
